# Lactic Acid Bacteria: A Promising Tool for Menopausal Health Management in Women

**DOI:** 10.3390/nu14214466

**Published:** 2022-10-24

**Authors:** Qian Chen, Haojue Wang, Gang Wang, Jianxin Zhao, Haiqin Chen, Xianyi Lu, Wei Chen

**Affiliations:** 1 State Key Laboratory of Food Science and Technology, Jiangnan University, Wuxi 214122, China; 2 School of Food Science and Technology, Jiangnan University, Wuxi 214122, China; 3 Department of Obstetrics and Gynecology, Wuxi Xishan People’s Hospital, Wuxi 214105, China; 4 National Engineering Research Center for Functional Food, Jiangnan University, Wuxi 214122, China; 5 Yangzhou Institute of Food Biotechnology, Jiangnan University, Yangzhou 225004, China

**Keywords:** lactic acid bacteria, menopause, gut microbiota, immune system, hormone replacement therapy

## Abstract

Menopause is a period during which women undergo dramatic hormonal changes. These changes lead to physical and mental discomfort, are greatly afflictive, and critically affect women’s lives. However, the current safe and effective management measures for women undergoing menopause are insufficient. Several probiotic functions of lactic acid bacteria (LAB) have been recognized, including alleviation of lactose intolerance, protection of digestive tract health, activation of the immune system, protection against infections, improvement of nutrient uptake, and improvement of the microbiota. In this review, we highlight the currently available knowledge of the potential protective effects of LAB on preventing or mitigating menopausal symptoms, particularly in terms of maintaining balance in the vaginal microbiota, reducing bone loss, and regulating the nervous system and lipid metabolism. Given the increasing number of women entering menopause and the emphasis on the management of menopausal symptoms, LAB are likely to soon become an indispensable part of clinical/daily care for menopausal women. Herein, we do not intend to provide a comprehensive analysis of each menopausal disorder or to specifically judge the reliability and safety of complementary therapies; rather, we aim to highlight the potential roles of LAB in individualized treatment strategies for the clinical management of menopause.

## 1. Introduction

The World Health Organization defines menopause as the permanent cessation of spontaneous menses, caused by the loss of ovarian follicular activity. Compounded by the effects of ageing, social and metabolic factors, daily activity, and impaired well-being, menopausal symptoms might be extremely severe and could significantly affect the quality of life of women [1]. As life expectancy is increasing, women tend to experience menopause for more than one-third of their lives, especially in western countries [2]. According to statistics, 25% of women experience severe menopausal symptoms [3]; these include central nervous system (CNS)-associated disorders and physical changes associated with metabolic changes, musculoskeletal changes, sexual and urogenital system dysfunction, and structural changes [4]. Some menopausal symptoms, for example, hot flashes, appear even begin in the perimenopause (a period that ovarian function changing until menstruation completely disappears) [5]. Having a healthy menopause has great social benefits. A healthy menopause is a dynamic condition in which permanent loss of ovarian function occurs; it is characterized by physical, psychological, and social self-satisfaction and may include illness and disability, and this enables women to achieve the desired level of resilience and self-management [6]. Menopause is inescapable; however, for women to live a healthy and happy life in the future, the alleviation of the adverse events that accompany menopause are particularly important. Hormone replacement therapy (HRT) is currently recognized as the most effective method for treating menopausal syndrome (e.g., vasomotor dysfunction and genitourinary syndrome). However, HRT is not suitable for all menopausal women and is not effective against chronic diseases such as diabetes or cognitive decline [7]. Non-hormonal strategies, including lifestyle changes, improved diet and nutriment, non-hormonal pharmaceuticals, and the adoption of behavioural and alternative medicine therapies, have also been proposed to manage menopausal symptoms [8]. Evidence suggests that lactic acid bacteria (LAB), particularly those used as probiotics, are effective in relieving complex menopausal disorders.

LAB are a group of microorganisms first recognized in the early 19th century [9]. LAB are Gram-positive, usually peroxidase-negative, mildly aerobic, acid-tolerant non-bacilli and cocci; they are found in various habitats, including the digestive tract, oral cavity, pneogaster, and vagina in humans and in ambient ecological niches, including processed dairy and animal products and plant products. LAB are generally regarded as beneficial microorganisms, and some LAB strains, including those belonging to the genera *Lactobacillus*, *Leuconostoc*, *Pediococcus*, *Bifidobacteria*, *Lactococcus*, and *Streptococcus*, are recognized as probiotics [10]. Probiotics are defined as “live microorganisms that confer health benefits on the host when taken in sufficient quantities” [11]. LAB could produce a variety of beneficial metabolites, including antimicrobial peptides, short-chain fatty acids, and lactic acid [11]. Hence, LAB-driven fermentation preserves biological activity and has a plethora of health-enhancing effects, such as improvement of food nutritional value, prevention of intestinal infections, suppression of antibiotic-associated diarrhoea, treatment of lactose intolerance, enhancement of immunomodulation, treatment of food allergies, anti-oxidant effects, anti-anxiety effects, enhancement of lipid metabolism, and suppression of tumours [12]. Furthermore, LAB regulate imbalances in the gut microbiota composition by increasing the beneficial bacterial population, enhancing intestinal epithelial barrier function, and regulating cytokine production [13]. While the health benefits of LAB and LAB-fermented foods have been reported in multiple studies and generalized in several reviews, in the present review, we focus on the benefits of LAB in women with menopausal symptoms.

## 2. Menopausal Symptoms

### 2.1. Anxiety and Depression

Compared with men, women are more susceptible to depression and anxiety, especially during periods of hormonal fluctuations, such as during the low oestrogen phase of the menstrual cycle, after childbirth, and during menopause [14]. A prospective community-based cohort study from China showed that the prevalence of depressive symptoms increased from 14.5% (premenopausal) to 18.2% (in transition) to 19.6% (postmenopausal) along the menopause trajectory, while the incidence of anxiety symptoms increased from 3.1% (premenopausal) to 7.0% (in transition) to 7.4% (postmenopausal) [15]. In addition, anxiety and depression have been linked to sleep disorders and vasomotor symptoms in postmenopausal women [16].

### 2.2. Urogenital Atrophy

Urogenital atrophy is a common issue during menopause. Oestrogen loss during urogenital atrophy leads to vaginal dryness, vulvovaginal irritation and pain, dyspareunia, and recurrent urinary tract infections in 50–60% of postmenopausal women [12].

### 2.3. Osteoporosis

Osteoporosis is a disease characterized by deteriorated bone integrity and strength and mainly occurs in women 10–15 years after menopause [13]. Postmenopausal women with osteoporosis are more likely to experience fractures [14]. Approximately 40% of all postmenopausal women are likely to experience an osteoporotic fracture during the course of their lives [15].

### 2.4. Cognitive Disorders

Nearly two-thirds of all women experience subjective cognitive difficulties during menopausal transition; these difficulties mainly manifest as forgetfulness, slow thinking, and inattention [16]. Furthermore, older women have a higher risk of Alzheimer’s disease than older men, suggesting that longevity is not the reason underlying the sex-related difference [17].

### 2.5. Cardiovascular Risk

Menopause is recognized as a special cardiovascular risk factor in women. Early menopause has been linked to an increased risk of cardiovascular disease [18]. A previous cohort study reported that women who experienced menopause before 50 years of age had higher risks of death and cardiovascular events than those who experienced menopause after 50 years of age [19].

### 2.6. Metabolic Disorders

A 10-year follow-up study demonstrated that postmenopausal women had a higher body fat mass, more visceral fat, a greater fat percentage, and more central fat accumulation than premenopausal women [20]. These changes in fat distribution in menopausal women enhance insulin resistance and thereby induce an exponential increase in the incidence of diabetes. This increase, in turn, increases the risks of cardiovascular disease and death in women [21]. It is signified that metabolic disorders during menopause not only increase the risk of cardiovascular disease in menopausal women but also increase the national health system burden and affect the socio-economic development of society [22,23]. Thus, metabolic disorders in menopausal women should be considered not only as personal issues but also as socioeconomic issues [24].

## 3. The Mainstream Method of Managing Menopause

Decline in oestrogen levels during menopause is associated with multiple diseases in women. The understanding of menopause is increasing as various perceptions and symptomatic experiences of menopause are being described; this has promoted the medicalisation of the management of menopause-related symptoms. A positive attitude and appropriate coping strategies are necessary for a healthy menopause. Hormonal and non-hormonal strategies have also been recommended for menopausal women.

### 3.1. HRT

HRT is the most practical method for the treatment of menopausal symptoms caused by oestrogen withdrawal. Therapeutic administration of oestrogen might eliminate almost all menopausal symptoms. HRT comprises a series of preparations of sex hormones (oestrogen alone or combined with progestogen) that could be administered orally, transdermally, intramuscularly, intranasally, subcutaneously, or locally (vaginally) [25]. Several menopausal symptoms, including vasomotor symptoms, sleep disorders, loss of sexuality, bone loss, depression, memory deficits, and cardiovascular diseases, have been shown to be resolved after HRT [26]. However, the use of HRT is not recommended over long periods or after several years following menopause as it may increase the risk of breast cancer and coronary heart disease in older women [27]. Some studies have suggested that HRT should be primarily used to prevent menopausal symptoms in younger menopausal women [28]. In addition, HRT should be avoided in women with breast or endometrial cancer, cardiovascular disease, thromboembolic disease, and active liver disease [29].

### 3.2. Non-Hormonal Therapy

Tibolone (TIB) is a selective tissue oestrogen activity regulator that activates hormone receptors in a tissue-specific manner. The different hormone receptor effects of TIB are determined by its three metabolites: 3α-hydroxytibolone (3α-OH-T) and 3β-hydroxytibolone (3β-OH-T) have oestrogenic effects and combine with oestrogen receptors in the breast and brain tissues to exert estrogenic effects, whereas δ4-tibolone (δ4-TIB) exhibits an affinity for progesterone and androgen receptors in the endometrium and vagina, thereby exerting progestin and androgenic properties. TIB has an ameliorative effect on several symptoms of perimenopause, including vasomotor symptoms, mood- and cognition-related symptoms, neurodegeneration, sexual health-related symptoms, and bone demineralisation [30].

Selective oestrogen receptor modulators (SERMs) act as oestrogen agonists or antagonists in a tissue-selective manner, depending on the target tissue [31]. Tamoxifen, toremifene, and raloxifene are the first three SERMs approved for clinical use [32]. Tamoxifen is the most commonly used SERM and acts as an oestrogen receptor antagonist in breast tissue but as a partial agonist in other tissues such as the endometrium and bone [32]. Although tamoxifen may treat osteoporosis and reduce the incidence of cardiovascular disease and treat breast cancer, its long-term usage unfortunately has side effects such as hot flashes and uterine cancer [33].

Selective serotonin re-uptake inhibitors (paroxetine, citalopram and venlafaxine) and serotonin–norepinephrine re-uptake inhibitors (venlafaxine) are beneficial in alleviating the frequency and severity of hot flashes during menopause [34].

### 3.3. Non-Pharmaceutical Treatments

#### 3.3.1. Phytoestrogens

Non-steroidal polyphenolic plant substances are collectively known as phytoestrogens; this class mainly includes flavonoids, lignans, and stilbenes, all of which have biological activity similar to that of natural oestrogens and exert anti-oestrogenic and pro-oestrogenic effects by binding to oestrogen receptors [35]. Thus, the use of phytoestrogens is commonly recommended for alleviating menopausal symptoms. A study reported that phytoestrogens reduce menopausal hot flashes, night sweats, and other urogenital menopausal symptoms [36]. However, one study also indicated a lack of conclusive evidence proving the effects of phytoestrogen on menopausal symptoms [37].

#### 3.3.2. Vitamin and Mineral Supplements

Vitamins might contribute to improving the quality of life in menopausal women. In particular, vitamins combined with minerals such as calcium have positive effects in perimenopausal and postmenopausal women [38].

## 4. Health-Promoting Benefits and Clinical Implications of LAB in Menopausal Women

### 4.1. LAB for Healthy Ageing

Menopause is a manifestation of ageing, and the occurrence of menopause accelerates the ageing process [39]. Thus, like in ageing, during menopause, a woman’s body inevitably undergoes immune changes and experiences oxidative stress. In 1996, Wise proposed that understanding and controlling changes in the ageing process could help develop strategies to mitigate the destructive effects of menopause [40]. As ovarian function declines, the levels of pro-inflammatory serum markers, including interleukin (IL)-1, IL-6, and tumour necrosis factor (TNF)-α increase, as does the body’s cellular response to these cytokines; moreover, the levels of cluster of differentiation (CD) 4-T cells and B-lymphocytes and the cytotoxic activity of natural killer cells decrease [41]. Elevated levels of proinflammatory cytokines in postmenopausal women reportedly contribute to the risk of chronic inflammatory disease [42]. LAB have been shown to prevent and effectively treat inflammatory bowel disease, mucositis, and even colon cancer through their immunostimulatory properties [43]. Several studies have reported that LAB modulate immunity via various mechanisms. LAB could interact with the gut-associated lymphoid tissue and thereby modulate host-immune responses [44]. For example, probiotic LAB could stimulate changes in the composition of the intestinal mucosa and influence the mucosal immune system, including inducing the change from T helper 2 (Th2) to T-helper 1 (Th1) responses, thereby promoting humoral immunity [45]. In addition, the consumption of LAB promotes intestinal barrier function by increasing mucus secretion, producing antimicrobial peptides to inhibit pathogenic growth, competing with pathogens for adhesion to the intestinal mucosa and increasing the expression of epithelial-cell-binding proteins [46].

Before menopause, adequate oestrogen serves as an antioxidant to balance oxidative stress [47]. During menopause, oestrogen production is erratic, and because of the loss of the antioxidant effects of oestrogen, reactive oxygen species are assumed to increase. Unfortunately, oxidative stress caused by the excess production of free radicals such as reactive oxygen species is a key contributor to the ageing process. Thus, levels of reactive oxygen species increase with ageing. Under the dual action of ageing and reactive oxygen species generation, the occurrence of menopausal symptoms is exacerbated. In addition to protecting the immune system, LAB respond to oxidative stress. Studies have reported that LAB have a good antioxidant capacity and protect against oxidative stress (Table 1). In vitro, many LAB strains and their metabolites have shown excellent scavenging capacity for 1,1-diphenyl-2-picrylhydrazyl, O_2_^−^, and H_2_O_2_ [48]. In addition, LAB resist oxidative stress by chelating metal ions such as Fe^2+^ and Cu^2+^. The chelation of metal ions may contribute more to the antioxidant capacity of LAB rather than to the superoxide dismutase activity, as reported by a previous study [49]. Furthermore, enzymatic regulation is another mode of action of LAB, including regulation of the auto-secretion of antioxidant enzymes, induction of the activity of host antioxidative enzymes, and regulation of certain reactive-oxygen-species-producing enzymes [50].

As a dietary intervention strategy, LAB treatment seems to have great application prospects in regulating the aging immune system, combating oxidative stress, ultimately restoring immunity, preventing infection, and enhancing healthy aging. To date, although several studies have reported the effect of LAB on aging, most researchers chose males in the study of the impact of LAB on the immune system of aging animals and the possible mechanisms [51,52]. However, there are gender differences in immune response. Gonadal hormones might have contributions to this sex differential. Therefore, it is necessary to conduct targeted research on the health effects of LAB on females in terms of aging and oestrogen withdrawal [53,54].

### 4.2. Promoting Oestrogen Receptor Response

The main biological effect of oestradiol is mediated by two intracellular receptors: oestrogen receptors α and β [63]. In addition to oestrogen, phytoestrogens interact with oestrogen receptors. The effects of phytoestrogens in the body are regulated by the gut microbiota; these microbes convert the phytoestrogens into bioactive substances that are more readily absorbed and have stronger oestrogenic and antiestrogenic properties [64]. Owing to structural differences in the intestinal microbiota, the bioavailability of phytoestrogens varies from person to person. LAB might promote the production of active substances in phytoestrogens by promoting the metabolism of phytoestrogens or by modulating the balance of the microbiota [65]. In recent years, several LAB strains, including *Lacticaseibacillus paracasei*, *Lactococcus garvieae*, *Ligilactobacillus salivarius*, *Lactobacillus gasseri*, and *Lactiplantibacillus plantarum* have been identified as possessing an excellent ability to secrete glycosidase and convert daidzein to equol [66]. Under suitable conditions of fermentation, *L. plantarum* 128/2 is fully biologically active against isoflavones in soymilk and possesses a remarkable capacity to produce β-glucosidase activity [67]. In addition to using fermentation to promote the biotransformation of isoflavones, the simultaneous consumption of LAB and isoflavones could increase the production of equol in the body. Isoflavone administration in conjunction with probiotic consumption increases equol production over time [68]. When administered with isoflavones, LAB not only optimise isoflavone bioavailability by directly affecting isoflavone metabolism but also indirectly provide health benefits by affecting other bacterial species, thereby enabling the optimisation of the performance of these phytoestrogens [69]. A series of studies have demonstrated that, with the help of LAB, phytoestrogens play a critical role in the health of menopausal women. The presence of *L. sporogenes* increases the intestinal absorption and bioavailability of soy isoflavones that appropriately and safely alleviate sleep disorders in postmenopausal women [70]. Soy milk fermented with *Lpb. plantarum* 1R1.3.2 rather than unfermented soy milk significantly decreased the serum osteocalcin concentration in menopausal women [71]. In addition to helping phytoestrogens to bind to oestrogen receptors more efficiently, LAB supplements may influence the oestrogen metabolism by regulating the oestrobolome. For example, β-glucuronidase secreted by microorganisms can activate conjugated oestrogen, induce its reabsorption, and subsequently act on the oestrogen receptor [72]. Moreover, several studies involving polycystic ovarian syndrome (PCOS) showed that sex-hormone-related gut microbiota was modulated after the administration of LAB, thereby restoring abnormal sex hormone levels [73,74]. Several studies have showed that LAB is directly or indirectly involved in the regulation of hypothalamic pituitary hormones [75,76]. This provides a possibility of using LAB to regulate the sex hormone levels in the host. A recent study has shown that *Lpb. plantarum* CCFM1180 increased the circulating oestradiol concentration in ovariectomised mice and upregulated the expression of oestrogen receptor α in the abdominal adipose tissue [77]. This study indicated that the regulatory effect of LAB on oestrogen synthesis in extragonadal tissues will be shown when female gonad tissue function is weakened, such as the withdrawal of ovarian function in women during menopause. This provides a new idea to improve the oestrogen deficiency in women during menopause through dietary regulation. Unfortunately, the research has not been able to explain the specific mechanisms that mediates the regulation of oestrogen by this strain. With the development of omics technology, the potential targets and specific mechanism of LAB regulating gonadal hormone can be explored in future research by combining metagenomics, metabolomics, transcriptomics, and other technical means.

### 4.3. Role of LAB on Menopausal Osteoporosis

**Figure 1:**
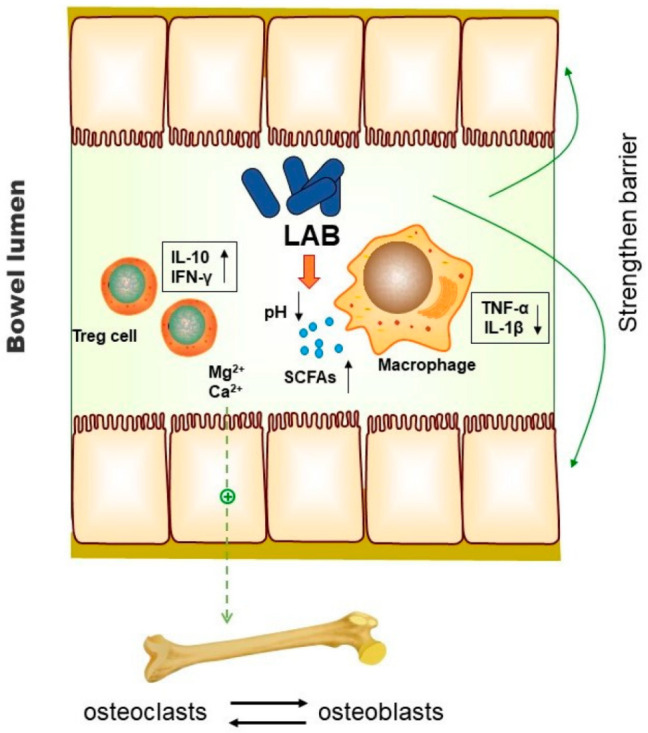
Possible mechanism of action of LAB on bone through the intestinal tract. Promotion of mineral release, enhancement of calcium absorption, inhibition of inflammation, and regulation of bone metabolism balance are the important mechanisms by which LAB inhibit osteoporosis by regulating the gut microbiota [81,82,83,84,85,86,87,88].

Bone homeostasis is maintained by the balance between bone resorption by osteoclasts and bone formation by osteoblasts [78]. The pathogenesis of osteoporosis is caused by bone resorption more than bone formation, resulting in a negative balance of bone metabolism [79]. Menopause results in a period of rapid bone loss, owing to oestrogen depletion. Higher consumption of yoghurt in older people is linked with higher bone density and greater physical function, attributable to the LAB it contains [80]. Numerous studies have shown that LAB could prevent bone resorption and bone loss (Table 2). The possible mechanism underlying bone protection by LAB regulating the intestinal microbiota has been proposed. As shown in Figure 1, LAB increase the availability of calcium by maintaining the low pH. In addition, the synthesis of vitamins and the production of short-chain fatty acids facilitated by LAB also result in increased calcium absorption [81]. Some active peptides produced by LAB can help release minerals from insoluble ions, thereby increasing mineral absorption [82]. LAB treatment acts on the immune system and attenuates increases in the osteoclastogenic factors (TNF-α and IL-1β) that are present during osteoporosis [83,84]. Regulatory T cells are protectors of bone health, and the consumption of LAB could regulate T cells to boost the transcription of the anti-osteoclastogenic factors interleukin (IL)-10 and interferon (IFN)-γ and thus enhance bone mass [85]. The lack of oestrogen during menopause leads to increased intestinal permeability [86]. Disruption of gut barrier integrity not only leads to gut inflammatory responses but also affects skeletal homeostasis. Strengthening the intestinal barrier function is one of the main roles of LAB [87]. Therefore, decreasing gut permeability is another potent mechanism of action by which LAB protect bone health during menopause [88]. Although oral LAB supplementation could be a potential alternative for preventing bone loss in menopause, more large samples and higher quality clinical trials are still needed to further verify the effectiveness of LAB in the intervention of osteoporosis or the maintenance of bone homeostasis. Moreover, considering that osteoporosis is a very common disease with a variety of causes, including lifestyle changes, genetic diseases, endocrine disorders, or the induction of other diseases, it is necessary to consider the impact of these factors on the effect of LAB intervention when designing clinical trials of menopausal osteoporosis. As shown in Table 2, in previous studies, the choice of experimental endpoint and different evaluation indicators will lead to incomparability between experimental results [83,89]. Therefore, more reliable and authoritative design and result measurement standards are needed for clinical trials targeting menopausal women with osteoporosis symptoms.

### 4.4. Prevention of Dyslipidaemia and Obesity by LAB

In the past few years, preclinical studies investigating the effects of LAB against the development of obesity have emerged. One study showed that *Lactobacillus* significantly reduces the feed intake by modifying the production of satiety hormones [93]. Treatment with *L. plantarum* CQPC02 reduced the visceral fat and blood lipid levels in obese mice consuming a high-fat diet. The lipid-lowering effects of *L. plantarum* CQPC02 may depend on its regulation of obesity-related mRNA expression in the liver [94]. Similarly, *L. plantarum* KFY04 slowed weight gain and decreased the weight of the liver and the visceral fat by modulating the expression of the peroxisome-proliferator-activated receptor pathway and by alleviating oxidative damage and inflammation in obese mice [95]. The gut microbiota plays a special role in energy absorption and metabolism. Firmicutes are more adept at metabolising energy than Bacteroides because they possess more genes for synthesising enzymes involved in lipid and carbohydrate metabolism [96]. Obese people often have a higher abundance of Firmicutes and a lower abundance of Bacteroidetes than lean people [97]. The modulation of the gut microbiota by LAB restores the integrity of gut function and exerts beneficial effects on the gut microbiota, reversing the dysregulated state of obesity [98]. Kefir, an LAB-fermented beverage, alleviated obesity and hepatic steatosis in mice consuming a high-fat diet by modulating the gut microbiota and mycobiota and suppressing inflammatory factors [99]. The effects of LAB on the regulation of metabolic disorders caused by oestrogen deficiency are also quite remarkable. A recent study showed that *L. intestinalis* YT2 alleviated the increased fat mass in ovariectomised rats mimicking the condition in menopausal women [100]. Moreover, a previous study showed that *Lpb. plantarum* CCFM1180 increased the level of circulating oestrogen, which may play a role in alleviating metabolic disorder in ovariectomised mice [36]. This evidence suggested that the mechanism of some LAB strains to alleviate metabolic diseases caused by oestrogen withdrawal may not be limited to their general regulation on the lipid metabolism. Meanwhile, this raises another question worthy of in-depth consideration, that is, whether the anti-obesity and anti-dyslipidaemia characteristics of strains in general metabolic disorder model will also be shown in the metabolic disorder model caused by changes in sex hormones. Although the mechanism of LAB regulating metabolic disorders in menopausal women is still unclear, a series of studies have been carried out to study the effects of LAB on metabolic disorders in menopausal women, and some progress has been made. A 12-week-long clinical study showed that multispecies probiotic supplementation inhibited risk factors in a dose-dependent manner and regulated glucose metabolism, blood lipid levels, waist circumference, and visceral fat in obese postmenopausal women [101]. Several current studies have shown that the dosage and duration of LAB supplementation affects efficacy of LAB in the treatment of lipid metabolism. Therefore, these factors need to be considered in the experimental design [102,103].

### 4.5. LAB and Their Influence on the Vaginal Microbiome

**Figure 2:**
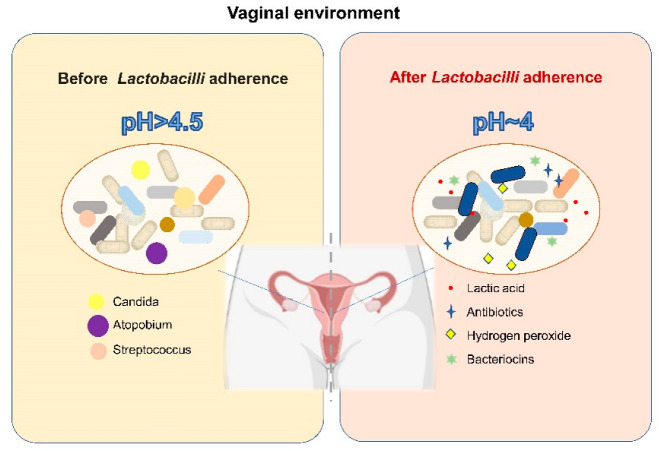
The mode of action of LAB on the vaginal microbiome [105,106,107,108,109,110,111].

The dominance of *Lactobacillus* among the microorganisms inhabiting the vagina of women receiving HRT has been reported previously; *Lactobacillus* is associated with the relief of vulvovaginal-atrophy-related symptoms and serum oestrone concentration [104]. Moreover, LAB play various protective roles in the vagina (Figure 2). First, *Lactobacillus* creates an acidic vaginal environment (pH = 4) by producing lactic acid, which has antibacterial properties [105,106]; in addition, LAB produce other antibacterial compounds, such as hydrogen peroxide, antibiotics, and target-specific bacteriocins, which may help promote the antagonistic ability of LAB and prevent foreign species from invading the vaginal microbiota [107,108,109]. Furthermore, *Lactobacillus* contributes to the ability of the microbiota to effectively exclude pathogenic microorganisms by modulating the epithelial innate immunity and competing for nutrients and tissue adherence [110,111]. Treatment with probiotics for vaginal dysbiosis is a relatively new concept. Several researchers have used probiotics to restore the *Lactobacillus* abundance in the vaginal microbiota of postmenopausal women and achieved promising results. In postmenopausal women treated with *L. rhamnosus* GR-1 and *L. reuteri* RC-14 (orally for 14 days), the vaginal flora was appropriately restored and the vaginal colonisation by potential pathogens and yeasts was reduced [111]. Moreover, it has also been reported that administration of live LAB strains in combination with low-dose oestriol has positive effects on restoring the vaginal microbiome and reducing urogenital atrophy and urinary tract infection [112,113,114]. Although recent scientific evidence shows that LAB used directly in the vagina and LAB introduced orally as adjuvant therapy may be a valuable strategy to restore the vaginal microbiota of menopausal women and protect them from genitourinary sequelae caused by oestrogen withdrawal, it should be pointed out that LAB used directly in the vagina and LAB introduced orally may play a role in the vagina through different modes and mechanisms, and researchers should put forward different requirements on the source and functional characteristics of LAB strains for oral or external use. Due to the need to ensure the outstanding ability of vaginal administrated LAB to colonize in the vagina and competitively exclude the pathogens, lactobacilli originating from the vagina of healthy women is the best candidate for LAB used directly in the vagina. In contrast, the sources of LAB introduced orally have more options. However, the difference of the mechanisms of LAB used directly in the vagina or LAB introduced orally on the vaginal microbiome and vaginal health remains unclear. Furthermore, questions remain as to how LAB introduced orally affect the vagina and how they migrate from the intestine to the vagina.

### 4.6. LAB and CNS-Related Symptoms

CNS-related symptoms are a result of neurobiochemical changes induced by ovarian function decline (e.g., vasomotor symptoms, somnipathy, anxiety and depression, migraine headaches, and cognitive impairment), and the symptoms are related [4]. It is important to control these symptoms in menopausal women. More recently, the term “psychobiotic” was linked to a series of probiotics that could influence the CNS [115]. LAB are effective in improving behaviours related to mental illness (Table 3). Indeed, some strains of psychobiotics, such as *L. plantarum* PS128^TM^ and *B. breve* CCFM1025, have been found to be effective against a variety of mental illnesses. LAB could secrete neuro-active metabolites that affect CNS-related symptoms. γ-Aminobutyric acid, which is generated by LAB, may delay the ageing of nerve cells, inhibit neurotransmitters, and mediate most inhibitory nerve transmissions in order to treat psychiatric disorders, including menopausal syndrome and early mental disorders [116]. In addition, LAB play critical roles in CNS-related symptoms by modulating the gut microbiota, inhibiting the working of the hypothalamic–pituitary–adrenal axis, regulating key neurotransmitters, strengthening the immune system, and modulating the neural system. Unfortunately, there is no sufficient evidence to directly prove that psychobiotics can improve the mood or psychology of menopausal women. It is recommended that further research is needed to assess the efficacy of LAB treatments on the relief of CNS-related symptoms in menopausal women. Moreover, considering the influence of changes in sex hormones and various menopausal symptoms on the CNS during menopause, the mode of action by LAB on CNS-related symptoms may involve other unspecified mechanisms.

## 5. Summary and Future Perspectives

When menopause occurs, women not only face problems related to ageing but also experience the adverse consequences of oestrogen withdrawal. Thus, the management of menopausal symptoms is very important. HRT is recognized as the most effective strategy to deal with menopausal symptoms; however, there are some concerns with its safety that limit its applicability. Additionally, HRT is not recommended for long-term usage. Thus, alternative therapies are needed for patients who are concerned about the safety of HRT or for those for whom HRT is not suitable. The strong evidence collected and reported in this review indicates that LAB supplementation has great prospects in the management of menopausal health. On the one hand, as a dietary intervention, LAB not only relay on their own salutary function to produce beneficial metabolites, fight oxidative stress, and enhance immunity but also manipulate the effects of the intestinal microbiota of the host via a two-way relationship with it. On the other hand, LAB that were thoroughly studied prior to application showed no adverse events during consumption, indicating that their use is safe. Therefore, when LAB are used as a mild dietary intervention, no risk level assessment is required.

Although LAB have many potential benefits for menopausal management, researchers still have a large amount of work to do, and extensive and systematic studies are necessary to demonstrate the effectiveness of LAB. Symptoms that occur during menopause, such as osteoporosis, depression, anxiety, and insomnia, are common. When designing a LAB intervention for women with menopausal symptoms, it is important not only to pay attention to the relief of general symptoms, but also to take into account whether LAB strains could address the root causes of the disease, such as menopausal sex hormone changes and immune aging. The regulation of LAB on the host’s gonadal axis, central nervous system, and immune system is not only related to the strain itself, but also may benefit from the gut microbiome and its metabolites regulated by LAB, such as short-chain fatty acids, bile acids, and amino acids. Therefore, genomics, metabolomics, transcriptomics, and other omics technologies should be fully combined to better understand the mechanism of LAB in relieving menopausal symptoms. Up until now, different standards have been adopted in clinical trials of LAB intervention for menopausal women. We suggest that the same standards should be adopted, and the experimental design should be based on authoritative guidelines, such as Good Clinical Practice guidelines of the International Council for Harmonization (GCP/ICH). Meanwhile, different endpoint judgment criteria should be designed for different menopausal symptoms. Furthermore, in order to improve the comparability between studies, clinical trial designers also need to consider the possible impact of diet, disease status, menopause, menstruation, fertility status, and even the race of menopausal subjects.

When adequately documented, we recommend that obstetricians and gynaecologists incorporate LAB interventions into perimenopausal and menopausal management strategies. After quantifying the symptoms and signs of menopause using standardized scales, the corresponding menopause management plan should be assigned to the patient according to the symptoms and severity. For those who want to enter menopause naturally, it is recommended that they supplement LAB therapy with immune protective, antioxidant, and mineral (e.g., calcium) supplements. LAB strains with symptom-targeted function may be recommended to prevent the recurrence of menopausal symptoms in those who have benefited from HRT and are ready to discontinue the treatment or reduce its dose. For patients on non-hormonal therapy, specific strains of LAB could be used in combination with medicines, such as selective serotonin re-uptake inhibitors, to alter tryptophan metabolism and relieve serious menopausal depressive symptoms.

## Figures and Tables

**Table 1:** Studies presenting evidence on the antioxidant effects of LAB (in vivo).

LAB Strain	Preclinical or Clinical Trials	Subjects	Sources of Oxidative Stress	Antioxidant Effects
*L. paracasei* BEJ01	Preclinical trials	Mice	Aflatoxin B1 and fumonisin B1	Reduced GSH concentration and inhibited expression of GPx and SOD [55]
*L. rhamnosus* GG	Preclinical trials	Mice	Colon and prostate cancer	Enhanced DPPH scavenging activity in colon and prostate cancer cells [56]
*L. lactis* subsp. *lactis* CCFM1018	Preclinical trials	Rats	DEHP	Inhibited the level of MDA and enhanced the level of CAT in DEHP-exposed rats [57]
*Lpb. plantarum* CCFM242	Preclinical trials	Mice	Ulcerative colitis	Decreased MDA content and increased the content of GSH, GPx, SOD, and CAT in the gut [58]
*P. pentosaceus* B19	Preclinical trials	Mice	PFOS	Enhanced GSH activity in the liver [59]
*L. casei* 01	Clinical trials	RA patients	RA	Decreased SOD and GPx activity [60]
*L. acidophilus* La5 and *B. lactis Bb*12	Clinical trials	MetS patients	MetS	Significantly increased the level of TAC [61]
*L. acidophilus*, *L. bulgaricus*, *L. bifidum*, and *L. casei.*	Clinical trials	Type 2 diabetes patients	Type 2 diabetes	Reduced MDA levels [62]

CAT: catalase; DEHP: di(2-ethylhexyl) phthalate; DPPH: α,α-diphenyl-β-picrylhydrazyl; GPx: glutathione peroxidase; GSH: glutathione; MDA: malondialdehyde; MetS: metabolic syndrome; RA: rheumatoid arthritis; SOD: superoxide dismutase; TAC: total antioxidant capacity.

**Table 2:** The regulatory effect of LAB on menopausal osteoporosis in preclinical trials or clinical trials.

LAB Strain	Preclinical or Clinical Trials	Subjects	Duration	Anti-Osteoporosis Results
*L. acidophilus*	Preclinical trials (in vitro)	MC3T3-E1 cells and RAW264.7 cells	-	Increased the number of cells in osteoblasts [90]
*Lpb. plantarum* NK3	Preclinical trials (in vivo)	Ovariectomized mice	4 weeks	Increased blood calcium, phosphorus, and osteocalcin levels [91]
*L. acidophilus* ATCC 4356	Preclinical trials (in vivo)	Ovariectomized mice	6 weeks	Strengthened both trabecular and cortical bone microstructure along with improved mineral density and heterogeneity of bones [85]
*L. reuteri* ATCC PTA 6475	Clinical trials (in vivo)	Elderly women with osteopenia	12 months	The loss in total VBMD of the distal tibia in women taking *L. Reuteri* 6475 was nearly half that of women taking placebo [92]
*L. paracasei* DSM 13434, *Lpb. plantarum* DSM 15312 and *Lpb. plantarum* DSM 15313	Clinical trials (in vivo)	Early postmenopausal women (lumbar spine: T score > −2.5)	12 months	Compared with placebo, LAB treatment reduced the LS-BMD loss [89]
Multispecies probiotic (Gerilact capsule)	Clinical trials (in vivo)	Postmenopausal women with mild bone loss	6 months	Decreased BALP and CTX levels by multispecies probiotic supplementation in comparison with the control group [83]

BMD: bone mineral density; LS-BMD: lumbar spine bone mineral density; OCN: osteocalcin; VBMD: volumetric bone mineral density.

**Table 3:** Evidence from animal experiments and human clinical trials of LAB treatment for improving mental symptoms.

Symptoms	Bacterial Strains	Subjects	Therapeutic Effects
Stress-related symptoms: anxiety, fatigue, sleep disturbance	*L. gasseri* CP2305	Healthy young adults	Reduced anxiety and fatigue and improved sleep quality, including shortened sleep latency and increased sleep duration [117]
Stress-related symptoms: anxiety, depression, sleep disturbance; Parkinson’s disease (PD); Alzheimer’s disease (AD)	*Lpb. plantarum* PS128TM	IT specialists PD patients AD mice	1. Apparently improved job and life satisfaction among IT specialists [118] 2. *Lpb. plantarum* PS128TM supplementation while continuously taking antiparkinsonian medicines could improve the motor ability of PD patients, inhibit the development of the disease, and improve the quality of life [119] 3. Effectively prevented damage in 3 × Tg-AD mice by ventricular injection of streptozotocin [120]
Depression disorder; Alzheimer’s disease	*B. breve* CCFM1025	Major depressive disorder patients AD mice	1. Significantly alleviated the psychiatric and gastrointestinal disorders of major depression disorder patients [121] 2. Significant improvement in cognitive impairment and neuroinflammation [122]

AD: Alzheimer’s disease; *B. breve*: *Bifidobacterium breve*; PD: Parkinson’s disease.

## Data Availability

Not applicable.

## References

[1] Honour J.W. (2018). Biochemistry of the menopause. Ann. Clin. Biochem..

[2] Lobo R.A., Gompel A. (2022). Management of menopause: A view towards prevention. Lancet Diabetes Endocrinol..

[3] Lee P.-S., Lee C.-L. (2020). Prevalence of symptoms and associated factors across menopause status in Taiwanese women. Menopause.

[4] Monteleone P., Mascagni G., Giannini A., Genazzani A.R., Simoncini T. (2018). Symptoms of menopause—Global prevalence, physiology and implications. Nat. Rev. Endocrinol..

[5] Greendale G.A., Lee N.P., Arriola E.R. (1999). The menopause. Lancet.

[6] Jaspers L., Daan N.M., van Dijk G.M., Gazibara T., Muka T., Wen K.-X., Meun C., Zillikens M.C., van Lennep J.E.R., Roos-Hesselink J.W. (2015). Health in middle-aged and elderly women: A conceptual framework for healthy menopause. Maturitas.

[7] Mehta J., Kling J.M., Manson J.E. (2021). Risks, benefits, and treatment modalities of menopausal hormone therapy: Current concepts. Front. Endocrinol..

[8] E Nappi R., Chedraui P., Lambrinoudaki I., Simoncini T. (2022). Menopause: A cardiometabolic transition. Lancet Diabetes Endocrinol..

[9] Stiles M.E., Holzapfel W.H. (1997). Holzapfel, Lactic acid bacteria of foods and their current taxonomy. Int. J. Food Microbiol..

[10] Garbacz K. (2022). Anticancer activity of lactic acid bacteria. Seminars in Cancer Biology.

[11] Sánchez B., Delgado S., Blanco-Míguez A., Lourenço A., Gueimonde M., Margolles A. (2017). Probiotics, gut microbiota, and their influence on host health and disease. Mol. Nutr. Food Res..

[12] Naumova I., Castelo-Branco C. (2018). Current treatment options for postmenopausal vaginal atrophy. Int. J. Women’s Health.

[13] Gosset A., Pouillès J.-M., Trémollieres F. (2021). Menopausal hormone therapy for the management of osteoporosis. Best Pract. Res. Clin. Endocrinol. Metab..

[14] Sadeghi H., Ashraf A., Zeynali N., Ebrahimi B., A Jehu D. (2021). Balance and functional mobility predict low bone mineral density among postmenopausal women undergoing recent menopause with osteoporosis, osteopenia, and normal bone mineral density: A cross-sectional study. Geriatr. Nurs..

[15] Sözen T., Özışık L., Başaran N.Ç. (2017). An overview and management of osteoporosis. Eur. J. Rheumatol..

[16] Greendale G.A., Karlamangla A.S., Maki P.M. (2020). The menopause transition and cognition. JAMA.

[17] Andrew M.K., Tierney M.C. (2018). The puzzle of sex, gender and Alzheimer’s disease: Why are women more often affected than men?. Women’s Health.

[18] El Khoudary S.R. (2020). Age at menopause onset and risk of cardiovascular disease around the world. Maturitas.

[19] Li Y., Zhao D., Wang M., Sun J.-Y., Liu J., Qi Y., Hao Y.-C., Deng Q.-J., Liu J., Liu J. (2021). Combined effect of menopause and cardiovascular risk factors on death and cardiovascular disease: A cohort study. BMC Cardiovasc. Disord..

[20] Razmjou S., Abdulnour J., Bastard J.-P., Fellahi S., Doucet É., Brochu M., Lavoie J.-M., Rabasa-Lhoret R., Prud’Homme D. (2018). Body composition, cardiometabolic risk factors, physical activity, and inflammatory markers in premenopausal women after a 10-year follow-up: A MONET study. Menopause.

[21] Woodward M. (2019). Cardiovascular disease and the female disadvantage. Int. J. Environ. Res. Public Health.

[22] Slopien R., Wender-Ozegowska E., Rogowicz-Frontczak A., Meczekalski B., Zozulinska-Ziolkiewicz D., Jaremek J.D., Cano A., Chedraui P., Goulis D.G., Lopes P. (2018). Menopause and diabetes: EMAS clinical guide. Maturitas.

[23] Meneses M.J., Silvestre R., Sousa-Lima I., Macedo M.P. (2019). Paraoxonase-1 as a regulator of glucose and lipid homeostasis: Impact on the onset and progression of metabolic disorders. Int. J. Mol. Sci..

[24] Kozakowski J., Gietka-Czernel M., Leszczyńska D., Majos A. (2017). Obesity in menopause–our negligence or an unfortunate inevitability?. Prz. Menopauzalny = Menopause Rev..

[25] Fait T. (2019). Menopause hormone therapy: Latest developments and clinical practice. Drugs Context.

[26] Vigneswaran K., Hamoda H. (2021). Hormone replacement therapy-current recommendations. Best Pract. Res. Clin. Obstet. Gynaecol..

[27] Pan M., Pan X., Zhou J., Wang J., Qi Q., Wang L. (2022). Update on hormone therapy for the management of postmenopausal women. BioScience. Trends.

[28] Chester R.C., Kling J.M., Manson J.E. (2018). What the Women’s Health Initiative has taught us about menopausal hormone therapy. Clin. Cardiol..

[29] Papadakis G., Hans D., Gonzalez-Rodriguez E., Vollenweider P., Waeber G., Marques-Vidal P.M., Lamy O. (2016). The benefit of menopausal hormone therapy on bone density and microarchitecture persists after its withdrawal. J. Clin. Endocrinol. Metab..

[30] Del Río J.P., Molina S., Hidalgo-Lanussa O., Garcia-Segura L.M., Barreto G.E. (2020). Tibolone as hormonal therapy and neuroprotective agent. Trends Endocrinol. Metab..

[31] Pinkerton J.V., Conner E.A. (2019). Beyond estrogen: Advances in tissue selective estrogen complexes and selective estrogen receptor modulators. Climacteric.

[32] Gómez-Coronado D., Lasunción M.A., Martínez-Botas J., Fernández-Suárez M.E. (2020). Role of cholesterol metabolism in the anticancer pharmacology of selective estrogen receptor modulators. Semin. Cancer Biol..

[33] Ahmad I. (2018). Tamoxifen a pioneering drug: An update on the therapeutic potential of tamoxifen derivatives. Eur. J. Med. Chem..

[34] Stubbs C., Mattingly L., A Crawford S., A Wickersham E., Brockhaus J.L., McCarthy L.H. (2017). Do SSRIs and SNRIs reduce the frequency and/or severity of hot flashes in menopausal women. J. Okla. State Med Assoc..

[35] Gorzkiewicz J., Bartosz G., Sadowska-Bartosz I. (2021). The Potential Effects of Phytoestrogens: The Role in Neuroprotection. Molecules.

[36] Chen L.-R., Ko N.-Y., Chen K.-H. (2019). Isoflavone Supplements for Menopausal Women: A Systematic Review. Nutrients.

[37] Saghafi N., Ghazanfarpour M., Sadeghi R., Najarkolaei A.H., Omid M.G., Azad A., Najarkolaei E.H. (2017). Effects of Phytoestrogens in Alleviating the Menopausal Symptoms: A Systematic Review and Meta-Analysis. IJPR.

[38] Vitale S.G., Caruso S., Rapisarda A.M.C., Cianci S., Cianci A. (2018). Isoflavones, calcium, vitamin D and inulin improve quality of life, sexual function, body composition and metabolic parameters in menopausal women: Result from a prospective, randomized, placebo-controlled, parallel-group study. Menopausal Rev..

[39] Lobo R.A. (2019). Menopause and aging. Yen and Jaffe’s Reproductive Endocrinology.

[40] Wise P.M., Krajnak K.M., Kashon M.L. (1996). Menopause: The aging of multiple pacemakers. Science.

[41] Fischer V., Haffner-Luntzer M. (2021). Interaction between bone and immune cells: Implications for postmenopausal osteoporosis. Seminars in Cell & Developmental Biology.

[42] Han A., Kim J.Y., Kwak-Kim J., Lee S.K. (2021). Menopause is an inflection point of age-related immune changes in women. J. Reprod. Immunol..

[43] Levit R., De Giori G.S., Leblanc A.D.M.D., Leblanc J.G. (2018). Folate-producing lactic acid bacteria reduce inflammation in mice with induced intestinal mucositis. J. Appl. Microbiol..

[44] Rajoka M.S.R., Shi J., Zhu J., Shao D., Huang Q., Yang H., Jin M. (2016). Capacity of lactic acid bacteria in immunity enhancement and cancer prevention. Appl. Microbiol. Biotechnol..

[45] Wang L., He Z., Tian P., Wang G. (2019). Lactic acid bacteria and host immunity. Lactic Acid Bacteria.

[46] LeBlanc J.G., Levit R., de Giori G.S., LeBlanc A.D.M.D. (2020). Application of vitamin-producing lactic acid bacteria to treat intestinal inflammatory diseases. Appl. Microbiol. Biotechnol..

[47] Sánchez-Rodríguez M.A., Zacarías-Flores M., Arronte-Rosales A., Correa-Muñoz E., Mendoza-Núñez V.M. (2012). Menopause as risk factor for oxidative stress. Menopause.

[48] Kuo H.-C., Kwong H.K., Chen H.-Y., Hsu H.-Y., Yu S.-H., Hsieh C.-W., Lin H.-W., Chu Y.-L., Cheng K.-C. (2021). Enhanced antioxidant activity of Chenopodium formosanum Koidz. by lactic acid bacteria: Optimization of fermentation conditions. PLoS ONE.

[49] Lee J., Hwang K.-T., Chung M.-Y., Cho D.-H., Park C.-S. (2005). Resistance of Lactobacillus casei KCTC 3260 to Reactive Oxygen Species (ROS): Role for a Metal Ion Chelating Effect. J. Food Sci..

[50] Feng T., Wang J. (2020). Oxidative stress tolerance and antioxidant capacity of lactic acid bacteria as probiotic: A systematic review. Gut Microbes.

[51] Ni Y., Yang X., Zheng L., Wang Z., Wu L., Jiang J., Yang T., Ma L., Fu Z. (2019). *Lactobacillus* and *Bifidobacterium* Improves Physiological Function and Cognitive Ability in Aged Mice by the Regulation of Gut Microbiota. Mol. Nutr. Food Res..

[52] Sharma R., Kapila R., Kapasiya M., Saliganti V., Dass G., Kapila S. (2014). Dietary supplementation of milk fermented with probiotic *Lactobacillus fermentum* enhances systemic immune response and antioxidant capacity in aging mice. Nutr. Res..

[53] Pennell L.M., Galligan C.L., Fish E.N. (2012). Sex affects immunity. J. Autoimmun..

[54] Moxley G., Posthuma D., Carlson P., Estrada E., Han J., Benson L.L., Neale M.C. (2002). Sexual dimorphism in innate immunity. Arthritis Care Res..

[55] Abbès S., Ben Salah-Abbès J., Jebali R., Ben Younes R., Oueslati R. (2015). Interaction of aflatoxin B_1_ and fumonisin B_1_ in mice causes immunotoxicity and oxidative stress: Possible protective role using lactic acid bacteria. J. Immunotoxicol..

[56] Celebioglu H.U. (2021). Effects of potential synbiotic interaction between Lactobacillus rhamnosus GG and salicylic acid on human colon and prostate cancer cells. Arch. Microbiol..

[57] Chen Q., Kong Q., Tian P., He Y., Zhao J., Zhang H., Wang G., Chen W. (2022). Lactic acid bacteria alleviate di-(2-ethylhexyl) phthalate-induced liver and testis toxicity via their bio-binding capacity, antioxidant capacity and regulation of the gut microbiota. Environ. Pollut..

[58] Zhai Q., Zhang Q., Tian F., Zhao J., Zhang H., Chen W. (2019). The synergistic effect of *Lactobacillus plantarum* CCFM242 and zinc on ulcerative colitis through modulating intestinal homeostasis. Food Funct..

[59] Chen Q., Sun S., Mei C., Zhao J., Zhang H., Wang G., Chen W. (2022). Capabilities of bio-binding, antioxidant and intestinal environmental repair jointly determine the ability of lactic acid bacteria to mitigate perfluorooctane sulfonate toxicity. Environ. Int..

[60] Vaghef-Mehrabany E., Rad A.H., Alipour B., Sharif S., Vaghef-Mehrabany L., Alipour-Ajiry S. (2015). Effects of Probiotic Supplementation on Oxidative Stress Indices in Women with Rheumatoid Arthritis: A Randomized Double-Blind Clinical Trial. J. Am. Coll. Nutr..

[61] Rezazadeh L., Alipour B., Jafarabadi M.A., Behrooz M., Gargari B.P. (2020). Daily consumption effects of probiotic yogurt containing Lactobacillus acidophilus La5 and Bifidobacterium lactis Bb12 on oxidative stress in metabolic syndrome patients. Clin. Nutr. ESPEN.

[62] Mazloom Z., Yousefinejad A., Dabbaghmanesh M.H. (2013). Effect of Probiotics on Lipid Profile, Glycemic Control, Insulin Action, Oxidative Stress, and Inflammatory Markers in Patients with Type 2 Diabetes: A Clinical Trial. Iran. J. Med Sci..

[63] Fuentes N., Silveyra P. (2019). Estrogen receptor signaling mechanisms. Adv. Protein Chem. Struct. Biol..

[64] Hameed A.S.S., Rawat P.S., Meng X., Liu W. (2020). Biotransformation of dietary phytoestrogens by gut microbes: A review on bidirectional interaction between phytoestrogen metabolism and gut microbiota. Biotechnol. Adv..

[65] Stojanov S., Kreft S. (2020). Gut Microbiota and the Metabolism of Phytoestrogens. Rev. Bras. Farm..

[66] Kwon J.E., Lim J., Kim I., Kim D., Kang S.C. (2018). Isolation and identification of new bacterial stains producing equol from Pueraria lobata extract fermentation. PLoS ONE.

[67] Izaguirre J., Barañano L., Castañón S., Alkorta I., Quirós L., Garbisu C. (2021). Optimization of the Bioactivation of Isoflavones in Soymilk by Lactic Acid Bacteria. Processes.

[68] Ribeiro A.E., Monteiro N.E.S., De Moraes A.V.G., Costa-Paiva L.H., Pedro A.O. (2018). Can the use of probiotics in association with isoflavone improve the symptoms of genitourinary syndrome of menopause? Results from a randomized controlled trial. Menopause.

[69] Monteiro N.E.S., Queirós L.D., Lopes D.B., Pedro A.O., Macedo G.A. (2018). Impact of microbiota on the use and effects of isoflavones in the relief of climacteric symptoms in menopausal women—A review. J. Funct. Foods.

[70] De Franciscis P., Grauso F., Luisi A., Schettino M.T., Torella M., Colacurci N. (2017). Adding Agnus Castus and Magnolia to Soy Isoflavones Relieves Sleep Disturbances Besides Postmenopausal Vasomotor Symptoms-Long Term Safety and Effectiveness. Nutrients.

[71] Desfita S., Sari W., Yusmarini Y., Pato U., Zakłos-Szyda M., Budryn G. (2021). Effect of Fermented Soymilk-Honey from Different Probiotics on Osteocalcin Level in Menopausal Women. Nutrients.

[72] Łaniewski P., Herbst-Kralovetz M.M. (2022). Connecting microbiome and menopause for healthy ageing. Nat. Microbiol..

[73] He Y., Wang Q., Li X., Wang G., Zhao J., Zhang H., Chen W. (2020). Lactic acid bacteria alleviate polycystic ovarian syndrome by regulating sex hormone related gut microbiota. Food Funct..

[74] Zhang J., Sun Z., Jiang S., Bai X., Ma C., Peng Q., Chen K., Chang H., Fang T., Zhang H. (2019). Probiotic *Bifidobacterium lactis* V9 Regulates the Secretion of Sex Hormones in Polycystic Ovary Syndrome Patients through the Gut-Brain Axis. mSystems.

[75] Poutahidis T., Springer A.D., Levkovich T., Qi P., Varian B.J., Lakritz J., Ibrahim Y.M., Chatzigiagkos A., Alm E.J., Erdman S.E. (2014). Probiotic Microbes Sustain Youthful Serum Testosterone Levels and Testicular Size in Aging Mice. PLoS ONE.

[76] Erdman S., Poutahidis T. (2014). Probiotic ‘glow of health’: It’s more than skin deep. Benef. Microbes.

[77] Chen Q., Wang B., Wang S., Qian X., Li X., Zhao J., Zhang H., Chen W., Wang G. (2021). Modulation of the Gut Microbiota Structure with Probiotics and Isoflavone Alleviates Metabolic Disorder in Ovariectomized Mice. Nutrients.

[78] Kim J.-M., Lin C., Stavre Z., Greenblatt M.B., Shim J.-H. (2020). Osteoblast-Osteoclast Communication and Bone Homeostasis. Cells.

[79] Li L., Wang Z. (2018). Ovarian aging and osteoporosis. Aging Aging-Relat. Dis..

[80] Binda S., Ouwehand A.C. (2019). Chapter 12: Lactic Acid Bacteria for Fermented Dairy Products. Lactic Acid Bacteria: Microbiological and Functional Aspects.

[81] Campbell J.M., Fahey J.G.C., Wolf B.W. (1997). Selected Indigestible Oligosaccharides Affect Large Bowel Mass, Cecal and Fecal Short-Chain Fatty Acids, pH and Microflora in Rats. J. Nutr..

[82] Matar C., Amiot J., Savoie L., Goulet J. (1996). The Effect of Milk Fermentation by Lactobacillus helveticus on the Release of Peptides During In Vitro Digestion. J. Dairy Sci..

[83] Jafarnejad S., Djafarian K., Fazeli M.R., Yekaninejad M.S., Rostamian A., Keshavarz S.A. (2017). Effects of a Multispecies Probiotic Supplement on Bone Health in Osteopenic Postmenopausal Women: A Randomized, Double-blind, Controlled Trial. J. Am. Coll. Nutr..

[84] Ohlsson C., Engdahl C., Fåk F., Andersson A., Windahl S.H., Farman H.H., Movérare-Skrtic S., Islander U., Sjögren K. (2014). Probiotics Protect Mice from Ovariectomy-Induced Cortical Bone Loss. PLoS ONE.

[85] Dar H.Y., Shukla P., Mishra P.K., Anupam R., Mondal R., Tomar G.B., Sharma V., Srivastava R.K. (2018). Lactobacillus acidophilus inhibits bone loss and increases bone heterogeneity in osteoporotic mice via modulating Treg-Th17 cell balance. Bone Rep..

[86] Collins F.L., Rios-Arce N.D., Atkinson S., Bierhalter H., Schoenherr D., Bazil J.N., McCabe L.R., Parameswaran N. (2017). Temporal and regional intestinal changes in permeability, tight junction, and cytokine gene expression following ovariectomy-induced estrogen deficiency. Physiol. Rep..

[87] Ren C., Zhang Q., de Haan B.J., Faas M.M., Zhang H., de Vos P. (2020). Protective effects of lactic acid bacteria on gut epithelial barrier dysfunction are Toll like receptor 2 and protein kinase C dependent. Food Funct..

[88] Li J.Y., Chassaing B., Tyagi A.M., Vaccaro C., Luo T., Adams J., Darby T.M., Weitzmann M.N., Mulle J.G., Gewirtz A.T. (2016). Sex steroid deficiency–associated bone loss is microbiota dependent and prevented by probiotics. J. Clin. Investig..

[89] Jansson P.-A., Curiac D., Ahrén I.L., Hansson F., Niskanen T.M., Sjögren K., Ohlsson C. (2019). Probiotic treatment using a mix of three Lactobacillus strains for lumbar spine bone loss in postmenopausal women: A randomised, double-blind, placebo-controlled, multicentre trial. Lancet Rheumatol..

[90] Chen C., Dong B., Wang Y., Zhang Q., Wang B., Feng S., Zhu Y. (2019). The role of Bacillus acidophilus in osteoporosis and its roles in osteocyte proliferation and differentiation. J. Clin. Lab. Anal..

[91] Kim D.E., Kim J.K., Han S.K., Jang S.E., Han M.J., Kim D.H. (2019). Lactobacillus plantarum NK3 and Bifidobacterium longum NK49 alleviate bacterial vaginosis and osteoporosis in mice by suppressing NF-κ B-Linked TNF-α expression. J. Med. Food.

[92] Nilsson A.G., Sundh D., Bäckhed F., Lorentzon M. (2018). Lactobacillus reuteri reduces bone loss in older women with low bone mineral density: A randomized, placebo-controlled, double-blind, clinical trial. J. Intern. Med..

[93] Belguesmia Y., Domenger D., Caron J., Dhulster P., Ravallec R., Drider D., Cudennec B. (2016). Novel probiotic evidence of lactobacilli on immunomodulation and regulation of satiety hormones release in intestinal cells. J. Funct. Foods.

[94] Zhao X., Zhang J., Yi S., Li X., Guo Z., Zhou X., Mu J., Yi R. (2019). Lactobacillus plantarum CQPC02 prevents obesity in mice through the PPAR-α signaling pathway. Biomolecules.

[95] Long X., Zeng X., Tan F., Yi R., Pan Y., Zhou X., Mu J., Zhao X. (2020). *Lactobacillus plantarum* KFY04 prevents obesity in mice through the PPAR pathway and alleviates oxidative damage and inflammation. Food Funct..

[96] Kallus S.J., Brandt L.J. (2012). The Intestinal Microbiota and Obesity. J. Clin. Gastroenterol..

[97] Cao S.-Y., Zhao C.-N., Xu X.-Y., Tang G.-Y., Corke H., Gan R.-Y., Li H.-B. (2019). Dietary plants, gut microbiota, and obesity: Effects and mechanisms. Trends Food Sci. Technol..

[98] Mazloom K., Siddiqi I., Covasa M. (2019). Probiotics: How effective are they in the fight against obesity?. Nutrients.

[99] Kim D.-H., Kim H., Jeong D., Kang I.-B., Chon J.-W., Kim H.-S., Song K.-Y., Seo K.-H. (2017). Kefir alleviates obesity and hepatic steatosis in high-fat diet-fed mice by modulation of gut microbiota and mycobiota: Targeted and untargeted community analysis with correlation of biomarkers. J. Nutr. Biochem..

[100] Lim E., Song E.-J., Kim J., Jung S., Lee S.-Y., Shin H., Nam Y.-D., Kim Y. (2021). *Lactobacillus intestinalis* YT2 restores the gut microbiota and improves menopausal symptoms in ovariectomized rats. Benef. Microbes.

[101] Szulińska M., Łoniewski I., van Hemert S., Sobieska M., Bogdański P. (2018). Dose-Dependent Effects of Multispecies Probiotic Supplementation on the Lipopolysaccharide (LPS) Level and Cardiometabolic Profile in Obese Postmenopausal Women: A 12-Week Randomized Clinical Trial. Nutrients.

[102] Szulińska M., Łoniewski I., Skrypnik K., Sobieska M., Korybalska K., Suliburska J., Bogdański P. (2018). Multispecies Probiotic Supplementation Favorably Affects Vascular Function and Reduces Arterial Stiffness in Obese Postmenopausal Women—A 12-Week Placebo-Controlled and Randomized Clinical Study. Nutrients.

[103] Zarezadeh M., Musazadeh V., Faghfouri A.H., Sarmadi B., Jamilian P., Jamilian P., Tutunchi H., Dehghan P. (2022). Probiotic therapy, a novel and efficient adjuvant approach to improve glycemic status: An umbrella meta-analysis. Pharmacol. Res..

[104] Mitchell C.M., Srinivasan S., Zhan X., Wu M.C., Reed S., Guthrie K.A., LaCroix A.Z., Fiedler T., Munch M., Liu C. (2017). Vaginal microbiota and genitourinary menopausal symptoms: A cross-sectional analysis. Menopause.

[105] Kumar N., Behera B., Sagiri S.S., Pal K., Ray S.S., Roy S. (2011). Bacterial vaginosis: Etiology and modalities of treatment-A brief note. J. Pharm. Bioallied Sci..

[106] Selle K., Klaenhammer T.R. (2013). Genomic and phenotypic evidence for probiotic influences of Lactobacillus gasseri on human health. FEMS Microbiol. Rev..

[107] Hillier S.L., Krohn M.A., Rabe L.K., Klebanoff S.J., Eschenbach D.A. (1993). The Normal Vaginal Flora, H2O2-Producing Lactobacilli, and Bacterial Vaginosis in Pregnant Women. Clin. Infect. Dis..

[108] Vallor A.C., Antonio M.A.D., Hawes S.E., Hillier S.L. (2001). Factors Associated with Acquisition of, or Persistent Colonization by, Vaginal Lactobacilli: Role of Hydrogen Peroxide Production. J. Infect. Dis..

[109] Ma B., Forney L.J., Ravel J. (2012). Vaginal Microbiome: Rethinking Health and Disease. Annu. Rev. Microbiol..

[110] McMillan A., Dell M., Zellar M.P., Cribby S., Martz S., Hong E., Fu J., Abbas A., Dang T., Miller W. (2011). Disruption of urogenital biofilms by lactobacilli. Colloids Surf. B Biointerfaces.

[111] Bisanz J.E., Seney S., McMillan A., Vongsa R., Koenig D., Wong L., Dvoracek B., Gloor G., Sumarah M., Ford B. (2014). A Systems Biology Approach Investigating the Effect of Probiotics on the Vaginal Microbiome and Host Responses in a Double Blind, Placebo-Controlled Clinical Trial of Post-Menopausal Women. PLoS ONE.

[112] Özkinay E., Terek M.C., Yayci M., Kaiser R., Grob P., Tuncay G. (2005). The effectiveness of live lactobacilli in combination with low dose oestriol (Gynoflor) to restore the vaginal flora after treatment of vaginal infections. BJOG Int. J. Obstet. Gynaecol..

[113] Uehara S., Monden K., Nomoto K., Seno Y., Kariyama R., Kumon H. (2006). A pilot study evaluating the safety and effectiveness of Lactobacillus vaginal suppositories in patients with recurrent urinary tract infection. Int. J. Antimicrob. Agents.

[114] Capobianco G., Wenger J.M., Meloni G.B., Dessole M., Cherchi P., Dessole S. (2013). Triple therapy with Lactobacilli acidophili, estriol plus pelvic floor rehabilitation for symptoms of urogenital aging in postmenopausal women. Arch. Gynecol. Obstet..

[115] Visñuk D.P., de Giori G.S., LeBlanc J.G., de LeBlanc AD M. (2020). Neuroprotective effects associated with immune modulation by selected lactic acid bacteria in a Parkinson’s disease model. Nutrition.

[116] Wang S., Chen P., Dang H. (2019). Lactic acid bacteria and γ-aminobutyric acid and diacetyl. Lactic Acid Bacteria.

[117] Nishida K., Sawada D., Kuwano Y., Tanaka H., Rokutan K. (2019). Health Benefits of Lactobacillus gasseri CP2305 Tablets in Young Adults Exposed to Chronic Stress: A Randomized, Double-Blind, Placebo-Controlled Study. Nutrients.

[118] Wu S.-I., Wu C.-C., Tsai P.-J., Cheng L.-H., Hsu C.-C., Shan I.-K., Chan P.-Y., Lin T.-W., Ko C.-J., Chen W.-L. (2021). Psychobiotic Supplementation of PS128TM Improves Stress, Anxiety, and Insomnia in Highly Stressed Information Technology Specialists: A Pilot Study. Front. Nutr..

[119] Lu C.S., Chang H.C., Weng Y.H., Chen C.C., Kuo Y.S., Tsai Y.C. (2021). The add-on effect of Lactobacillus plantarum PS128 in patients with Parkinson’s disease: A pilot study. Front. Nutr..

[120] Huang H.J., Chen J.L., Liao J.F., Chen Y.H., Chieu M.W., Ke Y.Y., Hsu C.-C., Tsai Y.-J., Hsieh-Li H.M. (2021). Lactobacillus plantarum PS128 prevents cognitive dysfunction in Alzheimer’s disease mice by modulating propionic acid levels, glycogen synthase kinase 3 beta activity, and gliosis. BMC Complement. Med. Ther..

[121] Tian P., Chen Y., Zhu H., Wang L., Qian X., Zou R., Zhao J., Zhang H., Qian L., Wang Q. (2021). Bifidobacterium breve CCFM1025 attenuates major depression disorder via regulating gut microbiome and tryptophan metabolism: A randomized clinical trial. Brain Behav. Immun..

[122] Zhu G., Zhao J., Zhang H., Chen W., Wang G. (2021). Administration of *Bifidobacterium breve* Improves the Brain Function of Aβ_1-42_-Treated Mice via the Modulation of the Gut Microbiome. Nutrients.

